# The aetiology, prevalence and morbidity of outbreaks of photosensitisation in livestock: A review

**DOI:** 10.1371/journal.pone.0211625

**Published:** 2019-02-27

**Authors:** Yuchi Chen, Jane C. Quinn, Leslie A. Weston, Panayiotis Loukopoulos

**Affiliations:** 1 School of Animal and Veterinary Sciences, Charles Sturt University, Wagga Wagga, New South Wales, Australia; 2 Graham Centre for Agricultural Innovation, Charles Sturt University and NSW Department of Primary Industries, Wagga Wagga, New South Wales, Australia; 3 School of Agriculture and Wine Science, Charles Sturt University, Wagga Wagga, New South Wales, Australia; 4 Melbourne Veterinary School, University of Melbourne, Werribee, Victoria, Australia; University of Illinois, UNITED STATES

## Abstract

**Background:**

Photosensitisation is a clinical condition occurring in both humans and animals that causes significant injury to affected individuals. In livestock, outbreaks of photosensitisation caused by ingestion of toxic plants are relatively common and can be associated with significant economic loss.

**Objectives:**

The agents that are most commonly implicated in outbreaks of photosensitisation have not been formally investigated on a global scale. To address this question, a systematic review of the literature was undertaken to determine the most common causative agents implicated in outbreaks of photosensitisation in livestock in Australia and globally, as well as the prevalence and morbidity of such outbreaks.

**Methods:**

A systematic database search was conducted to identify peer-reviewed case reports of photosensitisation in livestock published worldwide between 1900 and April 2018. Only case reports with a full abstract in English were included. Non peer-reviewed reports from Australia were also investigated. Case reports were then sorted by plant and animal species, type of photosensitisation by diagnosis, location, morbidity and mortality rate and tabulated for further analysis.

**Results:**

One hundred and sixty-six reports qualified for inclusion in this study. Outbreaks were reported in 20 countries. Australia (20), Brazil (20) and the United States (11) showed the highest number of peer-reviewed photosensitisation case reports from this analysis. Hepatogenous (Type III) photosensitisation was the most frequently reported diagnosis (68.5%) and resulted in higher morbidity. *Panicum* spp., *Brachiaria* spp. and *Tribulus terrestris* were identified as the most common causes of hepatogenous photosensitisation globally.

**Conclusions:**

Hepatogenous photosensitisation in livestock represents a significant risk to livestock production, particularly in Australia, Brazil, and the United States. Management of toxic pastures and common pasture weeds may reduce the economic impact of photosensitisation both at a national and global level.

## Introduction

Photosensitisation is a global health issue affecting domestic livestock production with numerous underlying aetiological causes [1]. Clinical photosensitisation occurs when photodynamic compounds accumulate in the skin, cornea, and/or mucoid membranes [2]. Any portion of the animal exposed to sunlight and lacking protective fleece, hair or pigmentation can develop lesions within minutes to hours of exposure [3]. Severity can range from mild erythema and oedema to severe necrosis and skin sloughing [2].

Photosensitisation can be classified into three major categories based on aetiology: primary (Type I), congenital (Type II), and hepatogenous or secondary (Type III) [4]. Primary (Type I) photosensitisation occurs when photocytotoxic compounds, or their photoactive metabolites, are present within peripheral tissues following ingestion or via local percutaneous absorption following direct dermal contact [5]. Congenital (Type II) photosensitisation is rare and caused by abnormal heme synthesis resulting in accumulation of photodynamic metabolites, including uroporphyrin, coproporphyrin, and protoporphyrin derivatives in the skin [2,6]. Hepatogenous (Type III) photosensitisation is by far the most common in animals [2] and is caused by accumulation of phytoporphyrin (also known as phylloerythrin) in dermal tissues [7]. Any aetiological agent that impairs hepatobiliary excretion, either by damage to the hepatocytes directly (hepatotoxicity), or by damage to the functionality of the bile ducts themselves (cholestasis), can cause accumulation of phytoporphyrin resulting in clinical signs of photosensitisation [8].

Many outbreaks of photosensitisation are sporadic or transient [2]. Incidence and prevalence of photosensitisation varies depending on location, distribution of causative plants or fungi [9,10], the nature of the farming system [11], and environmental conditions [12], and the resistance/susceptibility of the species and individual animals within the flock or herd [12–20]. In livestock, the vast majority of photosensitisation outbreaks are associated with ingestion of plants [4,9].

Some cases are complex with synergistic or additive effects caused by different aetiological factors, a hypothesis supported by the sporadic pattern of certain outbreaks and the difficulty of experimentally recreating photosensitisation outbreaks associated with some plant species or other causal agents in their own right [2,21–23]. For example, sporidesmin, a mycotoxin contained in the spores of the saprophytic fungus *Pithomyces chartarum*, is capable of causing hepatogenous photosensitisation [12,24], especially in New Zealand where approximately 95% *P*. *chartarum* are toxigenic [25]. Sporadic outbreaks involving *P*. *chartarum* have suggested that sporidesmin may accelerate the progress of the liver damage in animals [2], when neither the presence of liver damage or the fungus would have been sufficient individually to cause photosensitisation [12,25]. The broad range of plant species implicated in outbreaks of secondary photosensitisation, the possibility of synergistic or interactive effects with mycotoxins, or other toxic entities, and the relative dearth of information on their biochemical profile commonly makes confirmation of a definitive causal agent problematic.

Several reviews of the incidence and prevalence photosensitisation in livestock have been published [1,4,26–31]. However, these have either focused on summarisation of diagnostic criteria [1,8,27,31,32], emphasised particular aetiological agents or been restricted to certain geographical regions [3,4,26,28,33]. As such, although providing useful information to the clinician or epidemiologist, they are generally limited in their ability to determine outbreak patterns, the prevalence of the causative agents, their relationship to morbidity, and the mortality on a larger scale. The lack of a holistic approach to the study of photosensitisation therefore limits our understanding of its impact on livestock globally.

The objective of this study was, therefore, to review the global presentation of cases of photosensitisation in the scientific literature, published in the English language, and to determine potential trends in which causal agents worldwide that are most commonly associated with outbreaks of photosensitisation in livestock. A review of the literature, including both peer-reviewed and non peer-reviewed sources, was undertaken for a better understanding of photosensitisation across a number of key countries globally where a significant body of published case information could be obtained.

## Methods

### Database analysis, search, and selection criteria: Peer-reviewed articles

Eleven electronic databases [Pubmed, Web of Science, CAB Abstracts, Proquest, Sciquest, Trove, EthOS, BASE, Open Access and Dissertations, NDLTD, and the DART Europe E Theses Portal] were mined for peer-reviewed articles relating to cases of photosensitisation in livestock. All case reports were extracted regardless of year or language of publication. Searches included the period from 1900 to April 2018. Search keywords included ‘photosensitisation’ and / or ‘photosensitization’, ‘photodermatitis’, ‘facial eczema’, ‘geeldikkop’, ‘dikoor’, ‘plochteach’ or ‘alveld’ in the title. The Boolean operator “OR” was used to join terms. Language restriction was applied; articles published in languages other than English, or did not provide an abstract in English, were excluded from the search result. Reference lists of retrieved articles were reviewed to identify all relevant case studies or reports to maximise article retrieval. The PRISMA checklist is attached as Supporting Checklist.

The following selection criteria were then applied; the publication should: 1) be an outbreak report with photosensitisation as the main clinical sign or differential diagnosis; 2) relate to any species of livestock, including alpaca, camel, cattle, deer, donkey, goat, horse, llama, mule, pig, reindeer, sheep, and water buffalo; and 3) contain clinicopathological findings that confirm a diagnosis of photosensitisation. Quality assessment of individual peer-reviewed reports was not performed. In particular, an assumption made with respect to the peer reviewed articles was that the causal agent responsible for all reported outbreaks were correctly identified in the associated publication.

**Fig 1:**
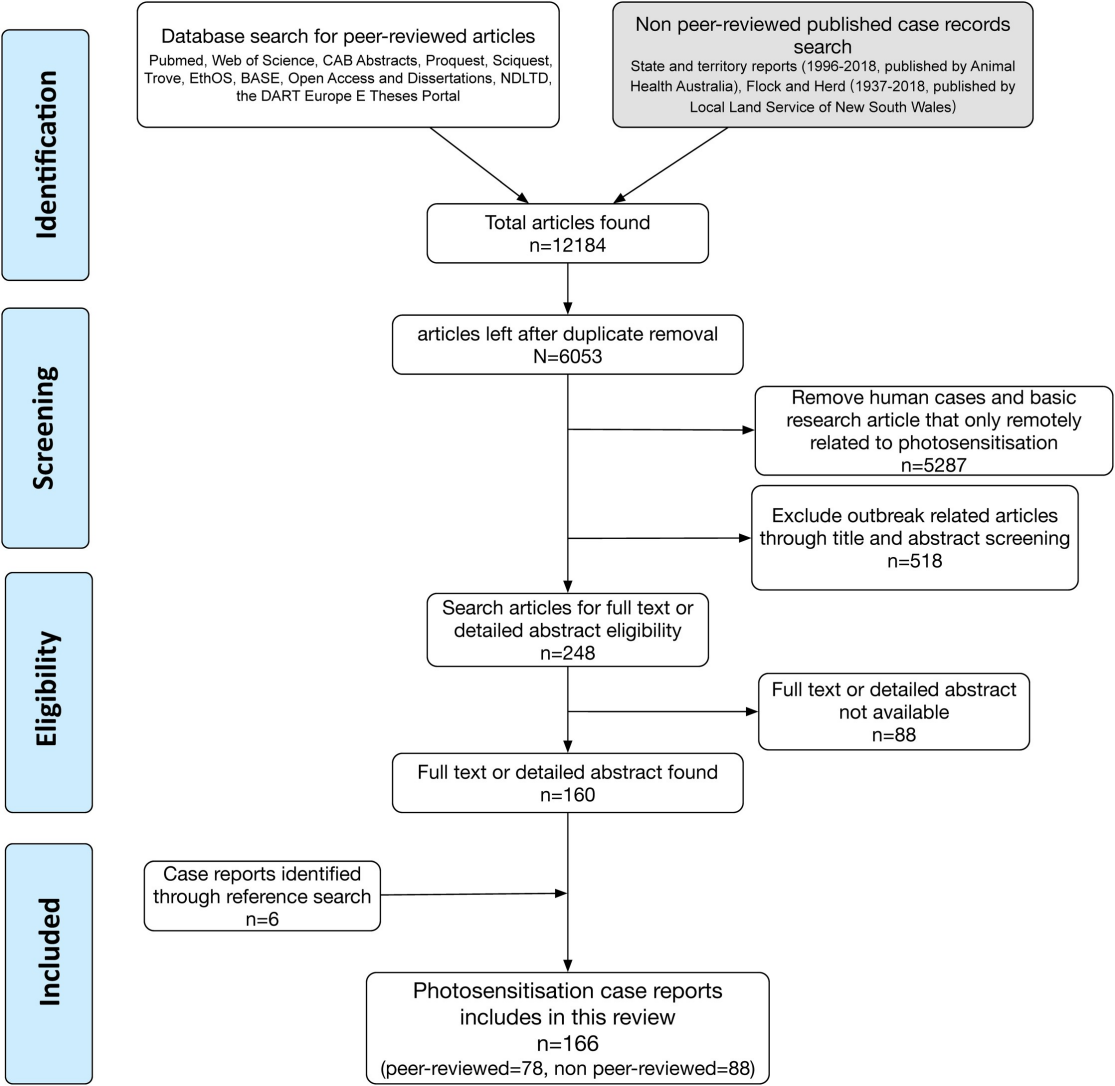
Literature search criteria and the number of articles included and excluded in this review.

Exclusion criteria included: duplications of published articles; species other than domestic livestock; publications in which the full text or a detailed abstract was not available; and publications in which the diagnosis of photosensitisation could not be established over other differentials. Search and selection methodology for this study is summarised in Fig 1.

### Database analysis, search, and selection criteria: Non peer-reviewed articles

Although the reliability of the diagnosis and the utility of information in non-peer reviewed reports can sometimes be questionable, it was considered that non peer-reviewed reports could be useful as supplemental information to peer-reviewed data by providing careful data extraction and thus review of the evidence in each report was undertaken. Australia was selected as the sole country for analysis of non peer- reviewed information due to its well-established case reporting systems via Australian Government Administrative or Government-Accredited Animal Health Officers (District Veterinarians, Regional Veterinary Officers, and Biosecurity Officers). Reports from these sources are published either in Animal Health Surveillance Quarterly Report (AHSQR, http://www.sciquest.org.nz/ahsq) that cover outbreaks nationally in Australia, or in the ‘Flock and Herd’ case note series that cover outbreaks in New South Wales (NSW) (F&H, http://www.flockandherd.net.au, site maintained by Local Land Services in NSW). Non peer-reviewed case material was searched up to and including April 2018. Again, the same selection and exclusion criteria were applied as described above.

Extracted information from non peer-reviewed case reports included, but was not limited to, species; age; quantitative and qualitative information regarding proportion of the herd or flock affected; geographical and chronologic information; type of photosensitisation and clinical description; causative agents confirmed or suspected. The accuracy of the diagnosis and entirety of the report was reviewed by the authors to ensure consistency, and reports, where clinicopathological data or causality did not appear consistent with a diagnosis of photosensitisation, were excluded from the analysis. However, due to the lack of peer-reviewed process, the accuracy and reliability of non peer-reviewed reports were considered to represent a lower level of evidence than peer-reviewed ones.

## Results

Following review of the peer-reviewed scientific literature, 78 reports presenting with a full text or detailed abstract were analysed (Fig 1). Data on causal plant species or organisms; geographical location; type of photosensitisation (primary, hepatogenous, congenital, unknown); outbreak years; animal species; size of flock or herd, percentage of morbidity and mortality were extracted and tabulated. A summary of information presented in the peer-reviewed literature is shown in Table 1.

**Table 1:** Extracted photosensitisation outbreak data from the peer-reviewed literature by aetiological agent. Percentage morbidity and mortality are included where reported.

Aetiological agent	Country	Type of photosensitisation	Year	Species	Flock/Herd size	Morbidity (%)	Mortality (%)	Reference
**Alfalfa hay** (predominantly *Medicago sativa*)	US	Primary	2013	Horse	116	6.9	N/A	[34]
**Alfalfa hay** (predominantly *M*. *sativa*)	US	Primary	2004	Horse	70	100.0	1.4	[34]
**Alfalfa hay** (predominantly *M*. *sativa*)	US	Primary	2008	Horse	N/A	N/A	N/A	[34]
***Alternanthera philoxeroides*** (Alligator weed)	AU	Hepatogenous	1998	Cattle	70	82.9	N/A	[35]
***Ammi majus*** (Bishop’s weed)	US	primary	1978	Sheep	N/A	N/A	N/A	[36]
***Biserrula pelecinus* vars. Casbah and Mauro** (Biserrula)	AU	Primary	2015	Sheep	167	100	N/A	[37]
***B*. *pelecinus* cv Casbah** (Biserrula)	AU	Primary	2013	Sheep	120	25.0	N/A	[38]
***Brachiaria brizantha*** (Palisade grass)	BR	Hepatogenous	2010	Sheep	80	16.3	12.5	[39]
***B*. *brizantha*** (Palisade grass)	BR	Hepatogenous	2010	Sheep	113	43.4	35.4	[39]
***Brachiaria decumbens*** (Signal grass)	CO	Hepatogenous	2015	Cattle	N/A	N/A	N/A	[20]
***B*. *decumbens*** (Signal grass)	MY	Hepatogenous	1985	Goat	12	25.0	N/A	[40]
***B*. *decumbens*** (Signal grass)	BR	Hepatogenous	2003	Goat	118	14.4	N/A	[41]
***B*. *decumbens*** (Signal grass)	BR	Hepatogenous	2003	llama	1	N/A	N/A	[42]
***B*. *decumbens*** (Signal grass)	BR	Hepatogenous	2009	Sheep	24	45.8	N/A	[21]
***B*. *decumbens*** (Signal grass)	NG	Hepatogenous	1982	Sheep	36	N/A	38.9	[43]
***B*. *decumbens*** (Signal grass)	BR	Hepatogenous	2009	Buffalo	17	52.9	N/A	[44]
***B*. *decumbens*** (Signal grass)	BR	Hepatogenous	2003	Sheep	28	25.0	21.4	[45]
***Brassica rapa*** (Turnip)	NZ	Hepatogenous	2014	Cattle	N/A	N/A	N/A	[46]
**Copper**	BR	Hepatogenous	2006	Buffalo	4	100.0	N/A	[47]
***Dicrocoelium dendriticum***	UK	Hepatogenous	2011	Sheep	65	49.2	3.1	[48]
***Enterolobium contortisiliquum*** (Pacara earpod tree)	BR	Hepatogenous	2014	Cattle	62	22.6	3.2	[49]
***E*. *contortisiliquum*** (Pacara earpod tree)	BR	Hepatogenous	2002	Cattle	N/A	N/A	N/A	[50]
**Flood damaged alfalfa hay** (predominantly *Medicago sativa*)	US	Hepatogenous	1957	Cattle	40	N/A	N/A	[51]
**Foxtail-or- chardgrass mixture cut hay**	US	Hepatogenous	1991	Cattle	8	100.0	12.5	[16]
***Froelichia humboldtiana*** (Ervanço)	BR	Primary	2014	Cattle	70	38.6	N/A	[52]
***F*. *humboldtiana*** (Ervanço)	BR	Primary	2014	Donkey	N/A	N/A	N/A	[53]
***F*. *humboldtiana*** (Ervanço)	BR	Primary	2014	Goat	15	100.0	N/A	[54]
***F*. *humboldtiana*** (Ervanço)	BR	Primary	2014	Mule	N/A	N/A	N/A	[53]
***F*. *humboldtiana*** (Ervanço)	BR	Primary	2006	Sheep	5	100.0	N/A	[55]
***F*. *humboldtiana*** (Ervanço)	BR	Primary	2014	Horse	N/A	N/A	N/A	[23] [53]
***Heliotropium europaeum*** (Common heliotrope)	AU	Hepatogenous	1985	Sheep	120	4.2	N/A	[13]
***Heracleum sphondylium*** (Hogweed)	UK	Primary	2010	Horse	N/A	N/A	N/A	[56]
***Hypericum erectum*** (St. John’s wort)	JP	Primary	1980	Cattle	5	100.0	N/A	[57]
***H*. *erectum*** (St. John’s wort)	TN	Primary	1999	Horse	34	N/A	N/A	[58]
***Jamesdicksonia dactylidis***	AU	Hepatogenous	2017	Cattle	678	24.3	2.8	[59]
**Liver fluke**	AT	Hepatogenous	2003	Cattle	N/A	N/A	N/A	[60]
***Lotus corniculatus*** (Birdsfoot trefoil)	NZ	Primary	1992	Sheep	40	7.5	N/A	[61]
***L*. *corniculatus*** (Birdsfoot trefoil)	NZ	Primary	1993	Sheep	56	26.8	N/A	[61]
***L*. *corniculatus*** (Birdsfoot trefoil)	NZ	Primary	1991	Sheep	30	33.3	N/A	[61]
***Malachra fasciata*** (Malachra)	BR	Primary	2016	Sheep	3	100	N/A	[62]
***Medicago sativa*** (Lucerne, alfafa)	ES	Primary	2004	Sheep	1850	24.3	N/A	[63]
***Microcystis aeruginosa***	SA	Hepatogenous	1993	Cattle	N/A	N/A	N/A	[64]
***Myoporum insulare*** (Common boobialla)	AU	Hepatogenous	1980	Cattle	177	14.1	6.2	[65]
***Narthecium ossifragum*** (Bog asphodel)	NO	Hepatogenous	1999	Sheep	165	9.7	N/A	[66]
***N*. *ossifragum*** (Bog asphodel)	NO	Hepatogenous	1990	Sheep	28	17.9	N/A	[18]
***Nodularia spumigena***	SA	Hepatogenous	1993	Cattle	N/A	N/A	N/A	[64]
***N*. *spumigena***	SA	Hepatogenous	1993	Sheep	N/A	N/A	N/A	[64]
**Pangola grass**	TW	Hepatogenous	1978	Cattle	8428	4.9	1.4	[67]
***Panicum coloratum*** (Klein grass)	US	Hepatogenous	1987	Sheep	24	100.0	N/A	[68]
***P*. *coloratum*** (Klein grass)	AU	Hepatogenous	1989	Sheep	2000	N/A	N/A	[69]
***Panicum dichotomiflorum*** (Fall panicum)	BR	Hepatogenous	2009	Sheep	365	22.2	10.7	[70]
***P*. *dichotomiflorum*** (Fall panicum)	US	Hepatogenous	2006	Horse	14	100.0	35.7	[71]
***Panicum miliaceum*** (Proso millet)	IR	Hepatogenous	2008	Sheep	10	10.0	N/A	[72]
***P*. *miliaceum*** (Proso millet)	IR	Hepatogenous	2008	Sheep	253	32.8	16.2	[73]
***Panicum schinzii*** (Sweet grass)	AU	Hepatogenous	1986	Sheep	200	30.0	20.0	[74]
***P*. *schinzii*** (Sweet grass)	AU	Hepatogenous	1991	Sheep	70	28.6	21.4	[75]
***P*. *schinzii*** (Sweet grass)	AU	Hepatogenous	1986	Sheep	200	25.0	15.0	[74]
***Panicum virgatum*** (Switch grass)	US	Hepatogenous	1991	Sheep	104	16.4	N/A	[76]
***Persicaria lapathifolia*** (Pale knotweed) ***and P*. *orientalis***	AU	Hepatogenous	2009	Cattle	50	4.0	20.0	[15]
***Petroselinum crispum*** (Parsley)	UK	Hepatogenous	1997	Pig	18	88.9	N/A	[77]
***Phytolacca octandra*** (Inkweed)	NZ	Hepatogenous	2006	Cattle	400	5.0	N/A	[78]
***Pithomyces chartarum***	NZ	Hepatogenous	1997	Fallow deer	20	60.0	30.0	[79]
***P*. *chartarum***	AU	Hepatogenous	1985	Sheep	200	15.0	N/A	[80]
***P*. *chartarum***	SA	Hepatogenous	1970	Sheep	N/A	N/A	N/A	[81]
***P*. *chartarum***	TR	Hepatogenous	2005	Sheep	1000	2.2	N/A	[82]
***P*. *chartarum***	US	Hepatogenous	1994	Sheep	N/A	N/A	N/A	[83]
***P*. *chartarum***	AU	Hepatogenous	1978	Sheep	22698	10.7	4.1	[84]
***Polygonum lapathifolium*** (Pale persicaria)	AU	Hepatogenous	1986	Cattle	380	N/A	1.6	[85]
**Porphyrins**	UK	CEP	2008	Cattle	N/A	N/A	N/A	[86]
**Porphyrins**	UK	CEP	1956	Cattle	N/A	N/A	N/A	[87]
**Protoporphyrin**	FR	CEPP	1991	Cattle	N/A	N/A	N/A	[88]
**Protoporphyrin**	IE	CEPP	2015	Cattle	20	5.0	N/A	[89]
**Protoporphyrin**	NZ	CEPP	2011	Cattle	N/A	N/A	N/A	[90]
**Protoporphyrin**	UK	CEPP	2000	Cattle	20	5.0	N/A	[91]
**Protoporphyrin**	UK	CEPP	2013	Cattle	26	7.7	N/A	[92]
**Protoporphyrin**	US	CEPP	1999	Cattle	70	1.4	N/A	[93]
***Senecio brasiliensis*** (Flor-das-almas)	BR	Hepatogenous	2013	Cattle	162	51.2	N/A	[94]
***Senecio spp***	BR	Hepatogenous	2014	Sheep	860	0.9	1.2	[95]
***Tribulus terrestris*** (Goat’s-head, puncture vine)	AU	Hepatogenous	1983	Goat	35	17.1	5.7	[96]
***T*. *terrestris*** (Goat’s-head, puncture vine)	AU	Hepatogenous	1982	Sheep	1200	20.8	14.7	[11]
***T*. *terrestris*** (Goat’s-head, puncture vine)	IR	Hepatogenous	1998	Sheep	11	100.0	N/A	[22]
***T*. *terrestris*** (Goat’s-head, puncture vine)	IR	Hepatogenous	1975	Sheep	700	8.5	4.3	[97]
***T*. *terrestris*** (Goat’s-head, puncture vine)	TR	Hepatogenous	2013	Sheep	24	100.0	N/A	[98]
***T*. *terrestris*** (Goat’s-head, puncture vine)	AU	Hepatogenous	1982	Sheep	190	36.8	24.2	[11]
***Trifolium alexandrinum*** (Berseem)	IN	Hepatogenous	2013	Cattle	N/A	N/A	N/A	[99]
**Unidentified**	MY	Hepatogenous	2012	Cattle	N/A	N/A	N/A	[100]
**Unidentified**	AU	Hepatogenous	1985	Sheep	35	42.9	28.6	[74]
**Unidentified**	AU	Hepatogenous	1986	Sheep	100	7.0	N/A	[74]
**Unidentified** (White clover, phalaris and rye grass)	AU	Hepatogenous	1964	Sheep	100	20.0	N/A	[101]

N/A, not available; CEPP, Congenital Erythropoietic Protoporphyria; CEP, Congenital Erythropoietic Protoporphyria; AU, Australia; AT, Austria; BR, Brazil; CO, Columbia; FR, France; IN, India; IR, Iran; IRE, Ireland; JP, Japan; MY, Malaysia; NZ, New Zealand; NG, Nigeria; NO, Norway; SA, South Africa; ES, Spain; TW, Taiwan; TN, Tunisia; TR, Turkey; UK, United Kingdom; US, United States

Following review of the Australian non peer-reviewed scientific literature, 88 non peer-reviewed Australian case reports with a full text or detailed abstract were identified for further review. Data on causal plant species or organism, country of outbreak, type of outbreak related to type of photosensitisation (primary, hepatogenous, congenital, unknown), animal species, size of flock or herd and percentage morbidity and mortality was extracted. Data contained in these reports was often less comprehensive than comparative peer-reviewed articles and information on the above criteria were extracted where available. A summary of information presented in Australian non peer-reviewed literature is shown in Table 2.

**Table 2:** Photosensitisation outbreaks in livestock in Australia extracted from two non peer-reviewed publication series. Percentage morbidity and mortality are included where available.

Aetiological agent	State	Type	Species	Flock/Herd size	Morbidity (%)	Mortality (%)	Reference
***B*. *decumbens*** (Signal grass)	WA	Hepatogenous	Sheep	300	20.0	N/A	AHSQR 17: 1
***Biserrula casbah*** (Biserrula)	WA	Primary	Sheep	500	40.0	N/A	AHSQR 7: 3
***Biserrula or clover***	WA	Primary	Sheep	N/A	N/A	N/A	AHSQR 9: 4
***Biserrula* spp.** (Biserrula)	WA	Primary	Cattle	N/A	N/A	N/A	AHSQR 16: 2
***Brassica napus*** (Rape, canola)	VIC	Hepatogenous	Cattle	200	3.0	N/A	AHSQR 14: 1
***Brassica* spp.**	VIC	Hepatogenous	Cattle	N/A	N/A	N/A	AHSQR 4: 1
***Cynosurus echinatus*** (Rough dog’s tail grass)	VIC	Hepatogenous	Cattle	9	N/A	22.2	AHSQR 19: 2
***C*. *echinatus*** (Rough dog’s tail grass)	VIC	Hepatogenous	Cattle	25	24.0	N/A	AHSQR 11: 2
***C*. *echinatus*** (Rough dog’s tail grass)	VIC	Hepatogenous	Cattle	35	28.6	N/A	AHSQR 11: 2
***C*. *echinatus*** (Rough dog’s tail grass)	VIC	Hepatogenous	Cattle	N/A	N/A	N/A	AHSQR 6: 2
***C*. *echinatus*** (Rough dog’s tail grass)	VIC	Hepatogenous	Cattle	510	32.4	10.0	AHSQR 7: 2
***C*. *echinatus*** (Rough dog’s tail grass)	WA	Hepatogenous	Cattle	N/A	N/A	N/A	AHSQR 7: 3
***C*. *echinatus*** (Rough dog’s tail grass)	VIC	Hepatogenous	Cattle	270	11.9	0.7	AHSQR 8: 2
***C*. *echinatus*** (Rough dog’s tail grass)	VIC	Hepatogenous	Cattle	11	100.0	N/A	AHSQR 15: 2
***C*. *echinatus*** (Rough dog’s tail grass)	VIC	Hepatogenous	Cattle	150	53.3	N/A	AHSQR 18: 2
***Echinochloa utilis*** (Japanese barnyard millet)	VIC	Hepatogenous	Sheep	300	10.0	6.7	AHSQR 14: 4
***Heliotrope* spp.**	VIC	Hepatogenous	Sheep	N/A	N/A	N/A	AHSQR 6: 3
***Heliotropium europaeum*** (Common heliotrope)	NSW	Hepatogenous	Cattle	60	100.0	66.7	F&H Sep, 2015
***H*. *europaeum*** (Common heliotrope)	NSW	Hepatogenous	Sheep	N/A	N/A	N/A	AHSQR 19: 4
***Hypericum perforatum*** (St John’s wort)	NSW	Primary	Sheep	550	33.6	N/A	F&H Mar, 2012
***H*. *perforatum*** (St John’s wort)	NSW	Primary	Sheep	300	50.0	3.3	AHSQR 13: 4
***Lantana camara*** (Lantana)	QLD	Hepatogenous	Cattle	100	N/A	3.0	AHSQR 11: 1
***L*. *camara*** (Lantana)	QLD	Hepatogenous	Cattle	35	5.7	2.8	AHSQR 12: 1
***L*. *camara*** (Lantana)	NSW	Hepatogenous	Cattle	N/A	N/A	N/A	AHSQR 14: 3
***L*. *camara*** (Lantana)	QLD	Hepatogenous	Cattle	250	N/A	3.2	AHSQR 16: 3
***L*. *camara*** (Lantana)	QLD	Hepatogenous	Cattle	250	0.8	N/A	AHSQR 16: 3
***L*. *camara*** (Lantana)	QLD	Hepatogenous	Cattle	20	50.0	N/A	AHSQR 16: 3
***L*. *camara*** (Lantana)	QLD	Hepatogenous	Cattle	80	N/A	7.5	AHSQR 21: 1
***L*. *camara*** (Lantana)	NSW	Hepatogenous	Cattle	47	N/A	19.2	AHSQR 8: 1
***L*. *camara*** (Lantana)	QLD	Hepatogenous	Cattle	120	N/A	2.5	AHSQR 10: 2
***Lantana* spp.**	NSW	Hepatogenous	Cattle	47	N/A	N/A	AHSQR 11: 4
***Lantana* spp.**	QLD	Hepatogenous	Cattle	N/A	N/A	N/A	AHSQR 12: 4
***Lantana* spp.**	QLD	Hepatogenous	Cattle	220	5.9	1.4	AHSQR 4: 2
***Lantana* spp.**	QLD	Hepatogenous	Cattle	800	18.8	N/A	AHSQR 7: 2
***Lantana* spp.**	QLD	Hepatogenous	Cattle	N/A	N/A	N/A	AHSQR 7: 3
***Lantana* spp.**	QLD	Hepatogenous	Cattle	N/A	N/A	N/A	AHSQR 9: 2
***Lolium perenne*** (Perennial ryegrass)	VIC	Primary	Cattle	120	25.0	N/A	AHSQR 12: 2
***Mentha pulegium*** (pennyroyal) ***and Lotus uliginosus*** (big trefoil)	TAS	Primary	Sheep	N/A	N/A	N/A	AHSQR 12: 1
***P*. *chartarum***	VIC	Hepatogenous	Cattle	23	69.6	30.4	AHSQR 6: 1
***P*. *chartarum***	WA	Hepatogenous	Cattle	750	40.0	N/A	AHSQR 8: 2
***P*. *chartarum***	NSW	Hepatogenous	Cattle	10	70.0	N/A	F&H Dec, 2011
***P*. *chartarum***	VIC	Hepatogenous	Cattle	N/A	50.0	‘significant’	AHSQR 4: 2
***P*. *chartarum***	SA	Hepatogenous	Cattle	N/A	N/A	N/A	AHSQR 5: 2
***P*. *chartarum***	TAS	Hepatogenous	Sheep	N/A	N/A	N/A	AHSQR 20:2
***P*. *chartarum***	VIC	Hepatogenous	Sheep & Cattle	114	5–50	1–30	AHSQR 16: 2
***P*. *chartarum***	WA	Hepatogenous	Sheep	N/A	N/A	N/A	AHSQR 9: 4
***P*. *chartarum***	NSW	Hepatogenous	Sheep	1000	N/A	N/A	F&H Nov, 2015
***P*. *effusum*** (Hairy panic), ***Brassica napus*** (Rape, canola) ***and Heliotropium europaeum*** (Common heliotrope)	VIC	Hepatogenous	Sheep	48	4.2	N/A	AHSQR 14: 1
***P*. *effusum*** (Hairy panic), ***T*. *terrestris*** (goat’s-head, puncture vine)	NSW	Hepatogenous	Sheep	230	100.0	N/A	AHSQR 14: 1
***P*. *gilvum*** (Sweet panic) ***and Brassica***	NSW	Hepatogenous	Sheep	500	8.4	N/A	AHSQR 18: 1
***P*. *coloratum*** (Klein grass)	NSW	Hepatogenous	Sheep	N/A	N/A	N/A	AHSQR 13: 1
***Panicum effusum*** (Hairy panic)	VIC	Hepatogenous	Sheep	350	14.3	8.6	AHSQR 15: 1
***Panicum gilvum*** (Sweet panic)	NSW	Hepatogenous	Sheep	520	7.7	1.2	F&H Jul, 2013
***Panicum hillmanii*** (Hillmann’s panic)	VIC	Hepatogenous	Sheep	400	6.3	N/A	AHSQR 14: 1
***Panicum miliaceum*** (Proso millet)	WA	Hepatogenous	Sheep	N/A	N/A	N/A	AHSQR 11: 3
***Panicum* spp.**	NSW	Hepatogenous	Sheep	many	N/A	N/A	F&H, 1981
***Panicum* spp.**	NSW	Hepatogenous	Sheep	N/A	N/A	10	AHSQR 1: 2
***Panicum* spp.**	VIC	Hepatogenous	Sheep	2170	N/A	1.4	AHSQR 20: 1
***Panicum* spp.**	VIC	Hepatogenous	Sheep	200	N/A	10.0	AHSQR 9: 1
***Panicum* spp.**	NSW	Hepatogenous	Sheep	450	5.1	N/A	F&H Sep, 2015
***Panicum* spp., *B*. *decumbens*** (Signal grass)**,*Chloris gayana*** (Rhodes grass), ***Echium plantagineum*** (Paterson’s curse)	WA	Hepatogenous	Cattle	500	2.0	N/A	AHSQR 15: 1
***Persicaria* spp.**	NSW	Hepatogenous	Cattle	50	4.0	N/A	F&H Apr, 2013
***Pithomyces chartarum***	TAS	Hepatogenous	Cattle	290	20.7	0.3	AHSQR 11: 1
***Pithomyces* spp.**	NSW	Hepatogenous	Sheep	14	14.3	N/A	AHSQR 16: 4
***Pithomyces* spp.**	WA	Hepatogenous	Sheep	1000	5.0	N/A	AHSQR 18: 2
***Polygonum* sp.**	NSW	Primary	Cattle	130	3.9	N/A	AHSQR 14: 4
***T*. *terrestris*** (Goat’s-head, puncture vine)	WA	Hepatogenous	Sheep	N/A	N/A	N/A	AHSQR 13: 1
***T*. *terrestris*** (Goat’s-head, puncture vine)	WA	Hepatogenous	Sheep	N/A	N/A	N/A	AHSQR 16: 3
***T*. *terrestris*** (Goat’s-head, puncture vine)	SA	Hepatogenous	Sheep	100	N/A	N/A	AHSQR 9: 1
***Tribulus terrestris*** (Goat’s-head, puncture vine)	NSW	Hepatogenous	Sheep	N/A	N/A	N/A	AHSQR 13: 1
***Trifolium resupinatum*** (Shaftal clover)	NSW	Hepatogenous	Sheep	N/A	N/A	N/A	F&H 1987
**Unidentified**	TAS	Primary	Cattle	150	3.3	N/A	AHSQR 12: 3
**Unidentified**	VIC	Hepatogenous	Cattle	300	6.7	N/A	AHSQR 15: 2
**Unidentified**	VIC	Hepatogenous	Cattle	250	20.0	N/A	AHSQR 15: 2
**Unidentified**	SA	Hepatogenous	Cattle	350	25.1	5.1	AHSQR 15: 2
**Unidentified**	VIC	Primary	Cattle	N/A	N/A	N/A	AHSQR 15: 3
**Unidentified**	TAS	Hepatogenous	Cattle	100	13.0	3.0	AHSQR 19: 4
**Unidentified**	TAS	Hepatogenous	Cattle	900	25.0	0.7	AHSQR 19: 4
**Unidentified**	TAS	Hepatogenous	Cattle	160	18.8	N/A	AHSQR 4: 2
**Unidentified**	WA	Primary	Sheep	N/A	N/A	N/A	AHSQR 16: 3
**Unidentified**	NSW	Primary	Sheep	N/A	50–100	N/A	AHSQR 20: 2
**Unidentified**	NSW	Primary	Sheep	8 flocks	50–100	5–15	F&H Nov, 2015
**Unidentified** (Aphid-infested thistles)	NSW	Hepatogenous	Cattle	36	100.0	41.7	F&H 1956
**Unidentified** (Aphids)	WA	Primary	Sheep	4200	N/A	N/A	AHSQR 12: 3
**Unidentified** (Pyrrolizidine alkaloids)	QLD	Hepatogenous	Horse	20	100.0	65.0	AHSQR 15: 2
**Unidentified** (Pyrrolizidine alkaloids)	SA	Hepatogenous	Sheep	N/A	N/A	N/A	AHSQR 13: 3
**Unidentified** (Ryegrass, silage, oats, straw, grape Marc and pasture hay)	VIC	Hepatogenous	Cattle	270	3.7	N/A	AHSQR 13: 1
**Unidentified** (Ryegrass)	VIC	Hepatogenous	Cattle	160	6.3	1.3	AHSQR 12: 2

N/A, not available; F&H, Flock and Herd, AHSQR, Animal Health Surveillance Quarterly; NSW, New South Wales; SA, Southern Australia; QLD, Queensland; TAS, Tasmania; VIC, Victoria; WA, Western Australia

### Geographical distribution and species differentiation of photosensitisation outbreaks reported in the peer-reviewed literature

**Fig 2:**
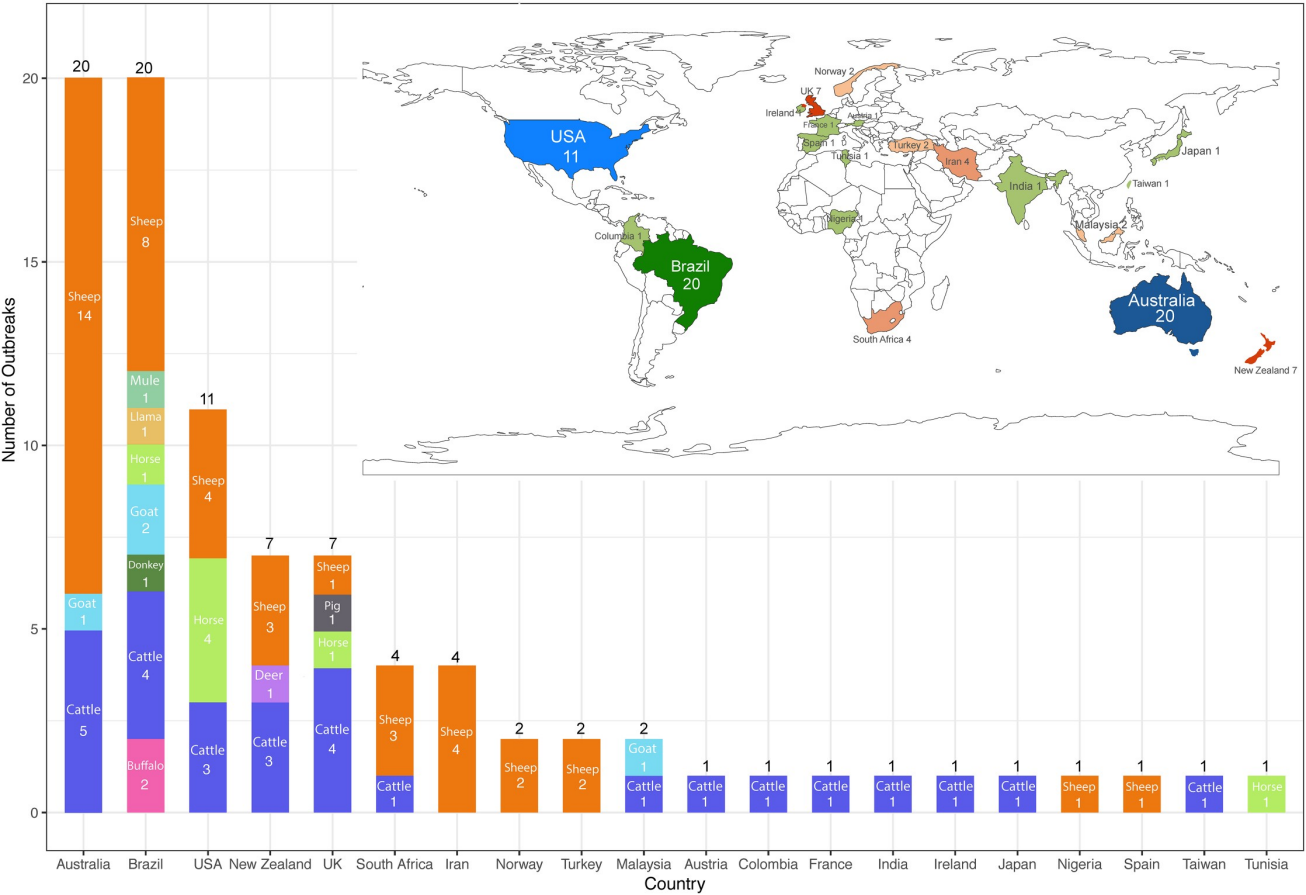
Global geographic distribution and species differentiation of peer-reviewed case reports of clinical photosensitisation in domestic livestock.

Photosensitisation outbreak reports were identified from 20 different countries in the peer-reviewed literature (Fig 2). The greatest number of reports were from Australia (20), Brazil (20), and the United States (11), followed by New Zealand (7), United Kingdom (7), South Africa (4), Iran (4), Norway (2), Turkey (2) and Malaysia (2), and 10 other countries with only one outbreak report each.

A prominent species predisposition was identified in these reports. Sheep were the most reported species in photosensitisation outbreaks globally, with 47.2% (42/89) reported outbreaks, followed by cattle (32.6%, 29/89) and horses (7.9%, 7/89). Other reported livestock species included the goat (4.5%, 4/89), buffalo (2.2%, 2/89), and one case each in the donkey, deer, mule, llama and pig (all 1.1%, 1/89).

### Geographical distribution and species differentiation of photosensitisation outbreaks reported in Australia

**Fig 3:**
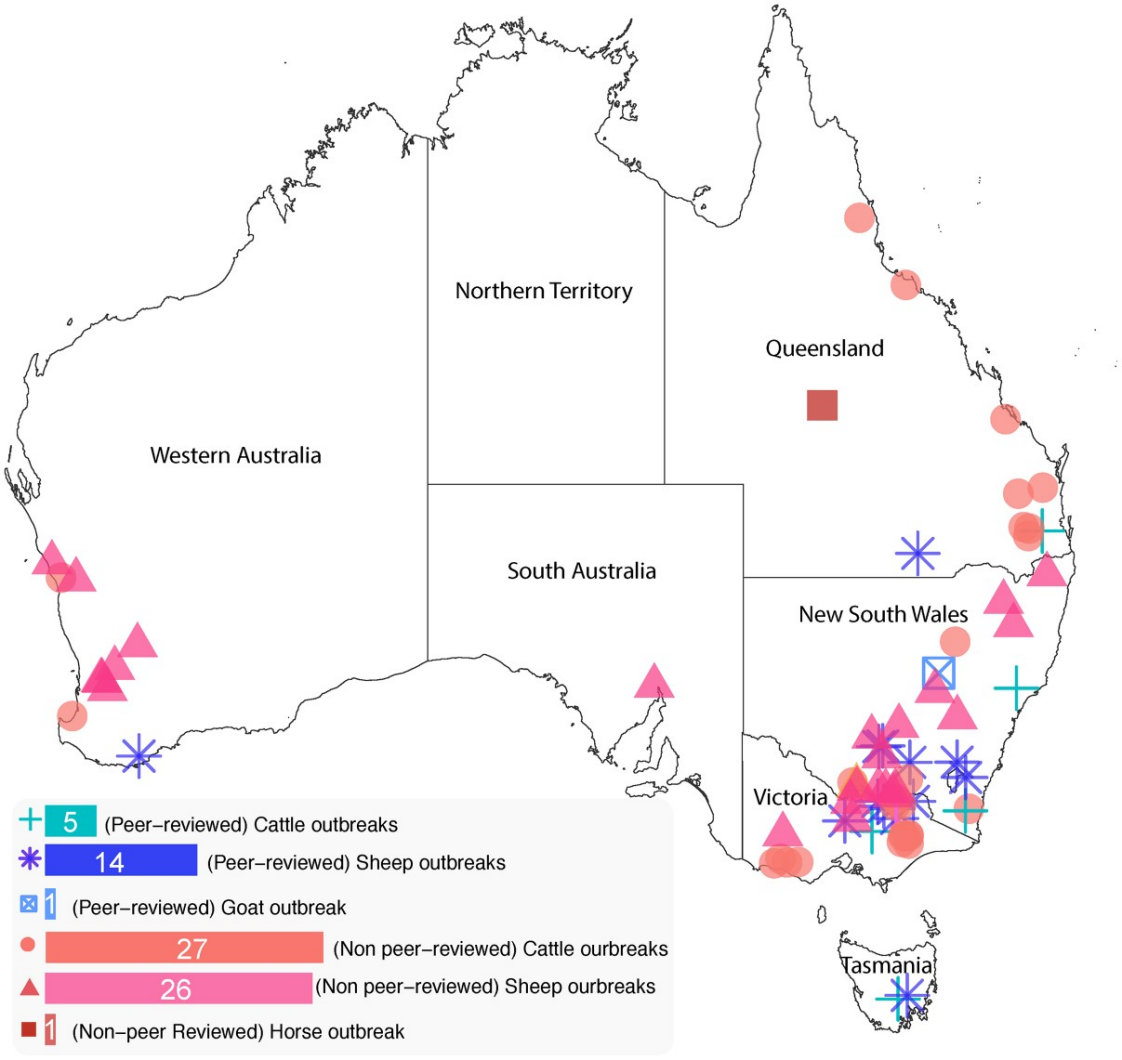
The number of combined peer-reviewed and non peer-reviewed photosensitisation case reports identified by geographical location and species in different states in Australia.

The geographical distribution of case data in Australia was further examined by state. When peer-reviewed and non peer-reviewed reports were considered together, the highest number of outbreaks was observed in New South Wales and Victoria (33 reports each), followed by Queensland (15 reports), Western Australia (15 reports), Tasmania (9 reports), and South Australia (4 reports) (Fig 3). There was no photosensitisation case report from the Northern Territory.

*Panicum* spp., *P*. *chartarum* and *T*. *terrestris* were clearly represented as causal agents with 12, 4, and 4 cases reported respectively. However, *Lantana* spp. (15 reports) and *Cynosurus echinatus* (9 reports) are also prevalent within specific geographical locations *(Lantana spp* in Queensland and *C*. *echinatus* in Victoria, Table 2).

As observed in global cases, a similar species predisposition, or reporting bias, was observed in reports from Australia. Sheep and cattle presented as the most frequently reported livestock species in photosensitisation outbreaks in Australia. Over 54% (40/74) of reported outbreaks concerned sheep, 43.2% (32/74) cattle, with the remaining outbreaks horse and goat (1 case only in each species).

### Prevalence of category of photosensitisation in livestock and aetiological agent, a global analysis

**Fig 4:**
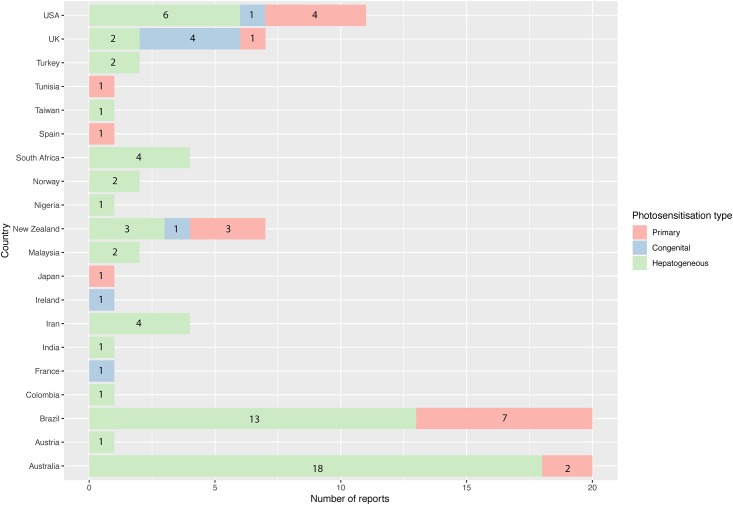
Numbers of peer-reviewed case reports by country by type of photosensitisation: Primary, congenital or hepatogenous.

In all peer-reviewed articles examined, 68.5% (61/89) reported cases were suspected or confirmed to be cases of hepatogenous (Type III) photosensitisation (Table 1). In comparison, only 22.5% (20/89) reported cases, were diagnosed or suspected to be primary (Type I) in nature. Only 9.0% (8/89) reported cases of outbreaks in the US, UK, New Zealand, Ireland, and France, were diagnosed or suspected as congenital (Type II) photosensitisation (Fig 4).

**Fig 5:**
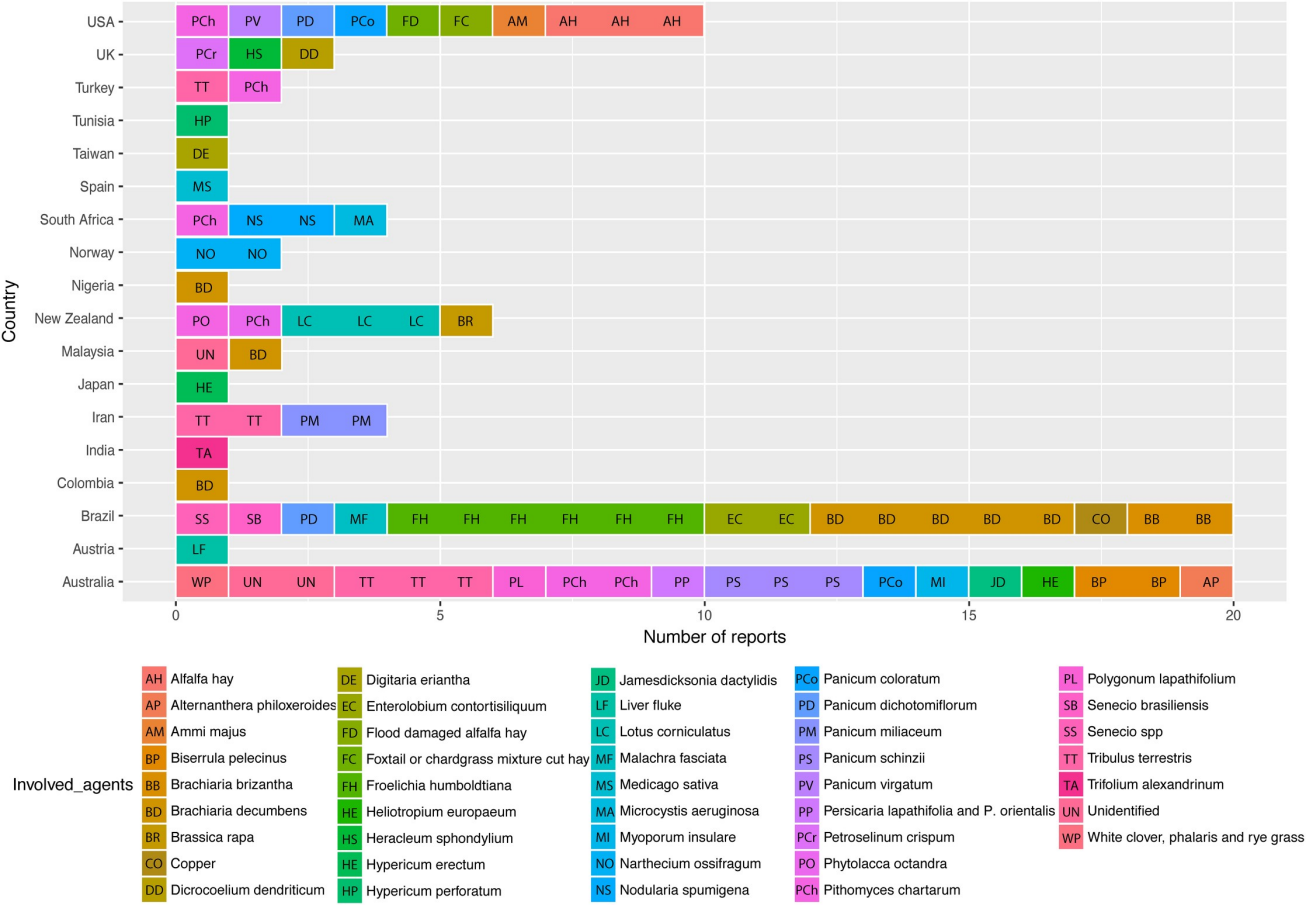
Causative agents identified in peer-reviewed photosensitisation case reports worldwide.

Common aetiological agents were reported in multiple locations globally (Fig 5). For example, *Brachiaria decumbens* has been reported as a causal agent in Brazil, Colombia, and Nigeria; various species of the *Panicum* genus of grasses have been identified as causal agents in outbreaks of photosensitisation in Australia, Brazil, Iran, and the United States; *P*. *chartarum*, a fungus that produces a specific mycotoxin called sporidesmin, was reported as an aetiological cause of hepatogenous photosensitisation in Australia, New Zealand, South Africa, Turkey, and the United States; *Tribulus terrestris* was reported as a causal species in Australia, Iran, and Turkey. Certain species were found to have a more contained geographical distribution, for example, *F*. *humboldtiana*, a primary photosensitising plant, was identified in six cases solely occurring in Brazil. *Biserrula* spp., an annual legume from the Mediterranean [102], was reported as a causative agent of primary photosensitisation only in Australia (Table 1, Fig 5).

Congenital cases of photosensitisation accounted for the least number of outbreaks which were restricted to the United Kingdom (4), France (1), Ireland (1), New Zealand (1), and the United States (1) (Table 1).

### Causative agents of photosensitisation in Australia

**Fig 6:**
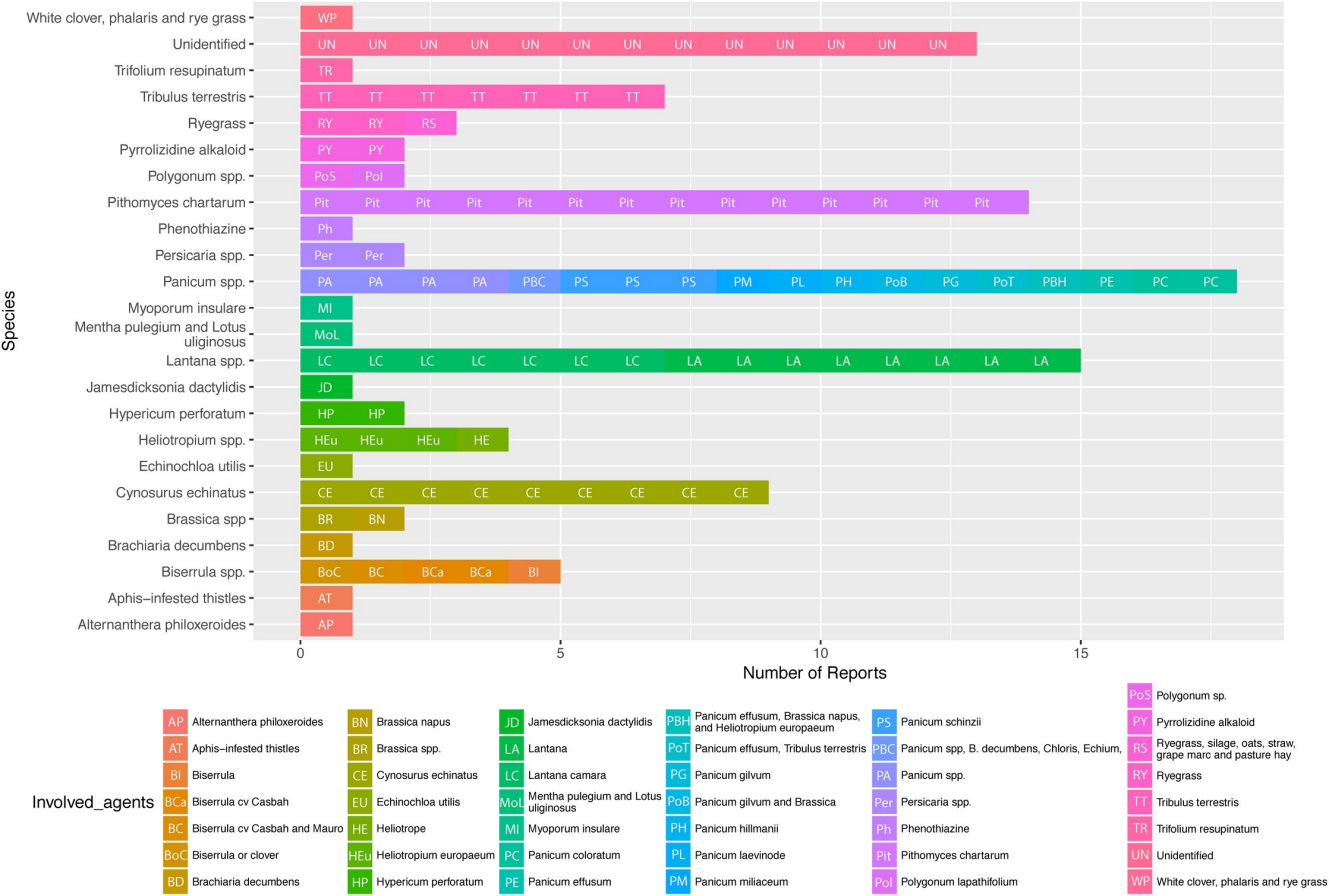
Plant species and other causative agents identified in published cases of photosensitisation in livestock in Australia.

Analysis of published photosensitisation case reports in Australia alone, taking both peer-reviewed and non peer-reviewed reports together, identified that outbreaks of photosensitisation in livestock related to ingestion of the *Panicum* genus of grasses were the most commonly reported aetiology in this region (18/94, 19.1%, Fig 6). The second most common confirmed aetiological agent was *Lantana* spp. (15/94, 16.0%). The soil-borne ubiquitous fungus *P*. *chartarum* also accounted for a significant proportion of analysed case reports (14/94, 14.9%). These three agents represented the confirmed aetiological causes of the majority of photosensitisation cases reported in Australia (47/94, 50%).

### Livestock morbidity related to photosensitisation type

**Fig 7:**
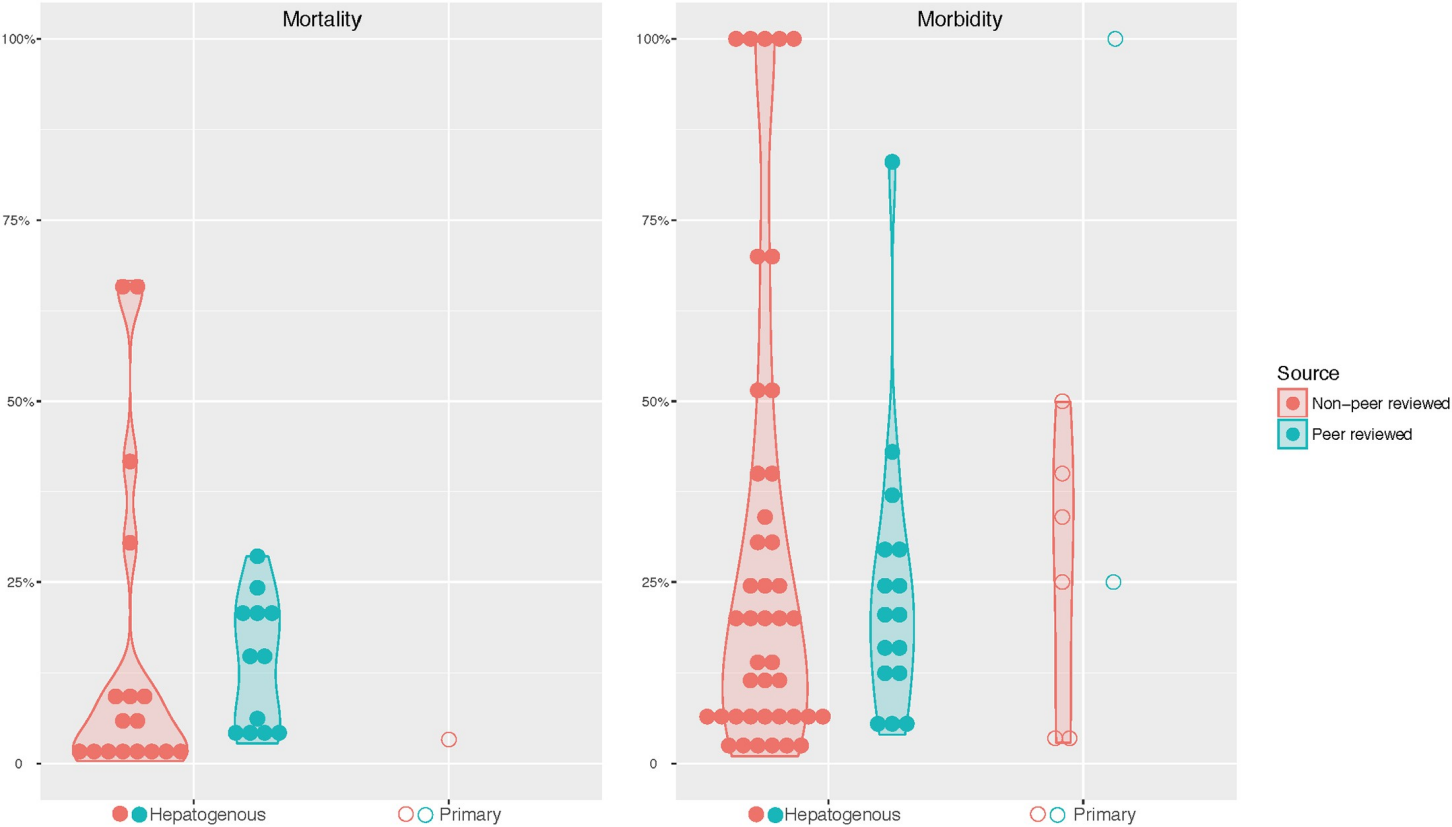
Mortality (a) and morbidity (b) in photosensitisation case reports published in the peer-reviewed and non peer-reviewed literature in Australia. Outbreaks reports that failed to provide an absolute number of the dead animal, the affected animal, or the flock size are not included.

Calculation of the morbidity (affected / flock number) and mortality (deaths / flock number) was compared between peer-reviewed reports (78 reports in total, Table 1) or non-peer reviewed reports (88 reports in total, Table 2) where the aetiological agent had been specified. The extracted data was visualised using a violin plot (Fig 7). This analysis showed a wide variation in morbidity and mortality between the two publication modalities with the peer reviewed publications showing higher figures for morbidity than those in non-peer reviewed publications. No photosensitisation related deaths were in outbreaks of congenital photosensitisation, and analysis showed that hepatogenous photosensitisation exhibited wider variation in morbidity and mortality than outbreaks of primary photosensitisation (Fig 7). Specifically, of the 26 peer-reviewed reports that described mortality related to photosensitisation, 25 were hepatogenous in nature. Only one mortality was associated with an outbreak of primary photosensitisation whilst none were associated with congenital cases (Fig 7a). Morbidity varied greatly ranging from 5–100% (Fig 7b).

## Discussion

### Photosensitisation outbreaks are commonly reported in Australasia, Brazil and the United States

Reporting bias was implicit in the method used for report extraction and data compilation in this study as only reports with a full text or abstract in English were selected for review and this fact explains, in part, the high numbers of case reports identified in Australia and the United States specifically. Each of these countries has a strong track record in scientific publication in the veterinary field, produces large numbers of sheep and cattle, also the most frequently reported livestock species in photosensitisation outbreaks (Table 1, Fig 2), and relies significantly on livestock production for export.

The number of reported cases from Europe as a whole was relatively low with only 12 articles found in the literature for this region. This finding suggests that photosensitisation is less of an issue for European farming systems compared to those of Australia, New Zealand, Brazil, and the US. Differences in climate [13–17], farming practices [12], availability of non-toxic pasture species and native or invasive weed species [103,104] associated with primary or secondary photosensitisation are likely to be playing a key role in this finding. However, the study selection criteria also would have been biased for publications from a large number of European countries as well. It possible that some photosensitisation outbreaks have been reported in native language publications, which would not have been selected due to by the filtering process. Nevertheless, the large number of case reports published in Brazil, identifying a diverse range of plant species as causally related to outbreaks of photosensitisation (Table 1, Fig 2), suggests that publication language barrier is not necessarily an obstacle to presentation of case reports in the literature, where English is the common language of scientific publication.

### Hepatogenous photosensitisation is common in domestic livestock

This report identifies hepatogenous photosensitisation to be the most common presentation of clinical photosensitisation in livestock (Fig 4), a finding in agreement with the general scientific literature [2]. Although inter-species and intra-species variation in sensitivity to photosensitising agents exists, toxic plants and organisms capable of causing outbreaks in domestic livestock should also be considered a potential risk to all grazing herbivores. This is particularly the case for native wildlife where plant-related outbreaks of hepatogenous photosensitisation have been reported in kangaroos and wombats [105–107].

Congenital (Type II) photosensitisation was rarely identified in the published literature (8/89 reports) in this study. Congenital erythropoietic protoporphyria has only been reported in cattle, and only in a small number of countries in Europe as well as the United States and New Zealand. This likely reflects the common ancestry of many cattle herds in aforementioned two countries with animals largely exported to these regions from the United Kingdom, and therefore the reliance on a small genetic pool of beef cattle in particular [90,93]. Interestingly, despite presenting with the highest number of peer-reviewed case reports, no congenital photosensitisation cases have yet been reported in Australia. This is likely a reflection of the selectivity of Australian livestock import systems and reliance on a highly conserved gene pool present in the cattle imported into this jurisdiction.

### Photosensitisation shows highly variable morbidity and mortality worldwide

A high degree of variability was reported for both morbidity and mortality in outbreaks of both primary and hepatogenous photosensitisation (Tables 1 & 2, Fig 7). This further suggests the difficulty of attributing an overall economic impact of photosensitisation to the global livestock industry, since the severity and magnitude of each outbreak is multifactorial and can differ significantly. An additional confounding factor is that higher mortality rates were recorded in hepatogenous photosensitisation found in peer-reviewed case reports compared to those reported in non-peer reviewed publications (Fig 7a), suggesting that only the most severe photosensitisation outbreaks were selected to be published by attending clinicians. This supports the author’s anecdotal findings that the majority of photosensitisation outbreaks are either submitted for publication in non peer-reviewed publications, or are not reported in print at all.

### Prevalence of photosensitisation case reports in Australia

Outbreak reports in this analysis represent a bias towards Australia. Certain countries, such as New Zealand, are also known anecdotally to have a high incidence of photosensitisation. The incidence of New Zealand may be underrepresented in this review as the available reporting systems for these outbreaks are not as apparent as Australia, where there is a strong network of government veterinarians and good mechanisms for the presentation of non peer-reviewed case reports. It is also widely acknowledged that the more common a disease, the less a producer is likely to request the services of a veterinarian for diagnoses, and the less a veterinarian is likely to report the outbreak formally.

Australia has experienced outbreaks of primary photosensitisation that are specific and unique to this region. One examples is photosensitisation caused by the pasture legume *Biserrula pelecinus*, although a native of the southern Mediterranean, it is exclusively used as a livestock fodder only in Australia [102] where this pasture legume is now clearly identified as causing outbreaks of primary photosensitisation [37,38]. Outbreaks that have never been recorded in its native domains where it grows as a native only in mixed swards. The specific need to identify drought tolerant, hardy annuals to fill the summer feed gap in Australian livestock production systems therefore resulted in introduction of pasture species that has selectively caused livestock toxicity [108]. This situation is not unique to Australia but this is the first clearly correlated example of an introduced species being propagated for pasture fodder which then gives rise to consistent outbreaks of primary photosensitisation in production livestock.

The majority of reports of photosensitisation cases in Australia reported in this review were located were in two states: New South Wales and Victoria (Fig 3). The disproportionate representation of these two states in this dataset is a reflection of a) the relatively high number of primary beef and lamb producers operating in these two states in Australia, and b) reporting bias due to availability of non-peer reviewed case reports presented in producer publications such as ‘Flock and Herd’ (a NSW publication). However, this relative over-reporting in our view does not rule out our findings that under-reporting of clinical outbreaks is still occurring in Australia due to the perception that photosensitisation is ’common’ in production flocks and herds (Y. Chen, survey of Australian veterinarians, unpublished data). Both states also showed a higher prevalence of causes related to *Panicum* genus of grasses *and P*. *chartarum*. Outbreaks in Queensland, by comparison, were mainly related to ingestion of the toxic weed species *Lantana* spp. These different causal species profiles suggest that environmental adaptation of such invasive plants is critical to their establishment and therefore the prevalence of toxicity associated with them [12,109].

An interesting finding that emerged from analysis of the non peer-reviewed Australian literature in this study was the identification of *C*. *echinatus* (rough dog’s tail) as a putative causative agent in outbreaks of hepatogenous photosensitisation from eight separate reports in Victoria and Western Australia (Table 2). Despite its common appearance in the non peer-reviewed literature, this plant has not been formally confirmed to be associated with an outbreak of photosensitisation [110] where controlled feeding trials have been unable to confirm *C*. *echinatus* as causing hepatotoxic damage sufficient to cause secondary clinical photosensitivity [111]. This contradictory evidence further suggests that anecdotal case reports should be viewed cautiously until true causality has been proven, as many epidemiological investigations focus on commonalities rather than specifics in outbreak patterns. In cases of photosensitisation a causal relationship between the clinical signs and the suspected agent(s) cannot be assumed to be proven until a direct or evidence-based causation can be established.

### Objective measurement of clinical signs of photosensitisation in domestic species

Morbidity in outbreaks of photosensitisation, of all underlying aetiological causes, was found to be highly variable in the literature. This variation is likely to be caused by reporting error by the producer or veterinarian based on inconsistent identification of affected animals. One issue that may have hampered accurate identification of the number of animals affected in outbreaks is the fact that mild cases are commonly overlooked by both the producer and veterinarian, therefore under-reporting of morbidity is likely to occur. Previously, no objective photosensitisation scoring protocol had been defined in literature resulting in prevalence and severity of affected animals to be hard to compare between outbreaks. Recently, a semi-objective photosensitisation grading system has been developed for sheep to address this issue [37]. The use of such a grading system in future outbreaks will allow better correlation between access to potentially photosensitising pastures or feedstuffs with more accurate determination of the timing of onset of outbreaks, as well as a more definitive and consistent identification of the severity of the condition. Furthermore, a mechanism for *de novo* case reporting related to known causal agents, not just those that are novel or unusual, (such as seen with the publication ’Flock and Herd’ or via an incidence register) would better document the prevalence, and therefore economic impact, of photosensitisation globally.

## Conclusions

Photosensitisation is a common, but likely underreported, entity in the literature. Hepatogenous photosensitisation is by far the most common presentation. Some species, the *Panicum* genus of grasses and *Pithomyces chartarum* in particular, consistently were reported in photosensitisation cases. Novel species are also implicated in outbreaks of photosensitisation, including pasture legumes *Biserrula pelecinus*, but primary photosensitisation is a rare occurrence in general. Significant variation in both morbidity, mortality and severity was observed in both peer-reviewed and non-peer reviewed reports. Variations in reported outbreaks may reflect true differences in morbidity rates between aetiological agents, but may also be partly due to the fact that mild presentations are overlooked, and lesions are not consistently graded by an unified standard. Together, our findings help identify the aetiology and geographical patterns, the plant and animal species implicated, and morbidity and mortality patterns of photosensitisation in livestock globally. This suggests that control of pasture species or weeds known to cause toxic outbreaks would have a significant impact on the prevalence of the condition in livestock globally, but also particularly in Australia, Brazil, and the United States where these outbreaks appear to be more common.

## Supporting information

S1 PRISMA Checklist (DOC)
